# Connections between trades and trafficking in wildlife and drugs

**DOI:** 10.1007/s12117-021-09416-z

**Published:** 2021-05-18

**Authors:** Daan van Uhm, Nigel South, Tanya Wyatt

**Affiliations:** 1 Utrecht University, Utrecht, The Netherlands; 2 University of Essex, Colchester, UK; 3 Northumbria University, Newcastle upon Tyne, UK

**Keywords:** Wildlife trafficking, Drug trafficking, Convergence, Green criminology, Criminal networks

## Abstract

Whilst drug trafficking has been a concern for several decades, wildlife trafficking has only fairly recently garnered international attention. Often media coverage of wildlife trafficking links it to the illegal trade of drugs. This article analyses wildlife and drug trafficking connections of various kinds. The purpose is to reveal the overlaps and synergies of wildlife and drug trafficking, providing concrete examples of where these markets co-exist as well as intertwine based on literature and original fieldwork. It explores the question of ‘Why in some cases, an illicit market remains focused on a single commodity, whilst in others it accommodates a combination of illicit commodities?’ This study identifies different types of wildlife-drugs linkages, including combined contraband, camouflage, multiple trade lines, shared smuggling routes and transportation methods, barter trade, and laundering drug money. The article shows that illicit markets are complex and the examples of activities and transactions that are provided illuminate some of the different dimensions of converging and diverging trades involving wildlife and drugs.

## Introduction

It is not uncommon for media coverage and policy discussions about the illegal wildlife trade to mention that it is linked to other forms of criminality, particularly drug trafficking (South and Wyatt 2011). Indeed, Massé et al. (2020) detail the exemplary case of such discussions during the 2018 London Wildlife Trade Conference. The illegal wildlife trade, they say, was defined as ‘serious organized crime’ and emphasis was placed upon its ‘convergence with other forms of “serious” crime’, such that a ‘common refrain’ was that criminal networks smuggling rhino horn and hardwood also traffic in drugs and guns. Massé et al. (2020: 30) argue that ‘little to no evidence was provided to support these statements’ and while there ‘is overlap between actors and networks trafficking in wildlife and other products, to suggest that this happens in every case … fails to … reflect the nuances of how harvesting and trade in some wildlife products occurs.’

In other words, overlaps do not always occur – and it is interesting to consider why not? Sometimes they do – but in varied and different ways. And the need to appreciate the complexity and ‘nuances’ of the practices, relationships and economics involved in all this is vital. Galemba (2017), Shelley (2018) and Mackenzie (2020), for example, explain how illicit trades may interact in varied ways, dependent upon the influences of globalization, new technologies and forms of communications.

In 2011, South and Wyatt (2011: 538) revisited their original fieldwork (Dorn and South 1990; Wyatt 2009) in order to examine ‘similarities and differences between two highly profitable, but distinctive illicit markets’. That paper sought to: further insights into the illegal wildlife trade through comparison with the more thoroughly studied illegal drug trade; to contribute to studies of drug trafficking by elaborating on its relationships with other forms of organized criminal enterprises; to draw together the fields of green criminology and the criminology of organized crime; and to aid in the development of prevention tactics. This paper builds upon this earlier exploratory framework, adds discussion of more recent published and grey literature,^1^ and presents original fieldwork that illustrates wildlife and drug trafficking connections of various kinds.

### Definitions

In this study, we are talking about drugs ‘with an illegal status in law’ and to a lesser degree legal drugs that are ‘diverted via theft or sale to an illicit market and sold and used illegally’ (South and Wyatt 2011: 542). Wildlife is defined as ‘all non-human animals, plants, and fungi which form part of a country’s natural environment or which are visitors in a wild or captured state’ and their derivatives (Nurse and Wyatt 2020: 5). Trade encompasses the collection, harvesting, possession, processing, acquiring, or transporting of wildlife for the purpose of purchasing, importing, exporting, selling, bartering, or exchanging. Wildlife trafficking or the illegal wildlife trade involves ‘the illegal trade, smuggling, poaching, capture, or collection of endangered species, protected wildlife (including animals or plants that are subject to harvest quotas and regulated by permits), derivatives, or products thereof’ (South and Wyatt 2011: 542).

### Aims

The purpose of this article is to revisit the possibilities of wildlife/drug trades overlaps and synergies, providing concrete examples of where these markets co-exist or intertwine. We explore the question ‘Why - in some cases - an illicit market remains focused on a single commodity, whilst in others it accommodates a combination of illicit commodities?’ One answer, as suggested by Reuter and O’Regan (2017) in their study of participants involved in ‘wildlife trafficking into the United States from elsewhere in the Western Hemisphere’ is that some individuals or small organisations are engaging with quite ‘distinct specialised markets’. In other cases, however, ‘[c]riminal organizations involved in wildlife trafficking are often directly connected to other trafficking networks, with the profits being used for all manner of illegal activities’ (C4, 2018). For instance, a 2018 U.S. Intelligence Community analysis of multi-agency crime data on an East African country found that ‘more than two-thirds of the actors in the wildlife crime dataset overlapped with individuals and facilitators in the narcotics dataset’ (TNRC 2020). Sosnowski (2019: 6) suggests that while Reuter and O’Regan ‘argue that there are more differences than commonly acknowledged between the illicit trades in wildlife and narcotics’ their study was ‘limited to trade activities in the Americas, and particularly smuggling into the United States.’ Sosnowski (2019: 6) points out that a more global view is required, and, for example, illegal ivory is ‘primarily sourced across Africa and directed to markets in Asia’ leading to different trade patterns which ‘closely mirror … narcotics trafficking.’

This paper also aims to do more to highlight the implications for species and environmental harms caused by both trades. All economic activity is ultimately based on the value of resources, and the subsequent production and trade of goods, legal or illegal, is dependent on processes of cultivation and harvesting, mining and extraction, manufacturing and distribution. These activities obviously result in legacies and consequences for environments and the non-human and human species affected. The paper explores these harms through some of the examples given. We begin by providing the background as to an overlooked importance of studying the illegal wildlife and drugs markets and their convergence. We then provide a brief overview of the two illegal markets. Then, we detail the methodology used to provide evidence of convergence and/or connections. This is followed by proposed categories of methods and motives operating within and linking these markets, and reference to evidence for these based on original empirical data as well as the research literature. The categories are: combined contraband, camouflage, multiple trade lines, shared smuggling routes and transportation methods, barter trade, and laundering for drug money. We then speculate as to how and why these methods and motives have emerged.

## Background

Although wide-ranging in scope, the original paper of South and Wyatt in 2011 was described by the authors as modest in ambition and clearly recognized the need for further research and for new ways of understanding and representing the complexity of the connections between these trades. In one respect, by signalling an aim to add to the growing literature on green criminology, it suggested how this might be achieved, but perhaps it did not make sufficiently clear why this was important. The most telling illustration of this is found in work by commentators (whether supportive of, or sceptical about, a proposition that there is any convergence between the trades and markets), when they report the numerous ways in which varieties of wildlife – whether reptiles, birds, fish, mammals or timber - provide concealment for drugs, but fail to note and explore what this reductionist utilisation and/or commodification of wildlife means or why this is important as an issue related to the intrinsic value of wildlife. As Ledec and Goodland (1988: 14) observe, ‘nonhuman species have their own intrinsic value independent of any practical or utilitarian value they may have for human beings’. For instance, Reuter and O’Regan (2017: 7) describe a case of smuggled live boa constrictors which ‘had been stuffed with condoms full of cocaine; the 312 snakes all died when the cocaine was retrieved.’ As the authors note ‘This is not a case of smuggling wildlife *and* drugs, but rather using the cover of legal wildlife imports as a method for concealing drugs’ (emphasis added). However, this does not note the further important point that this is also a case of animal abuse in which these reptiles are reduced to the status of simply being ‘containers’ for smuggling.

From a criminological point of view, the overlaps in smuggling, and mirroring or mingling of markets, are important areas of activity to review, but a *green* criminology also emphasises the threats to non-human species and environments (land, water) in creating and smuggling drugs and wildlife as commodities. As the respected journal *New Scientist* reported some years ago, ‘the narcotics trade is a serious but largely neglected impediment to conservation efforts’ (Aldhous 2006: 6) and drug production in remote areas, as well as drug eradication programs using chemical agents and militarised law-enforcement responses, will all be damaging to sensitive ecosystems and biodiversity. Such areas of biodiversity are also, of course, major sources of supply of traded wildlife. Furthermore, green criminology interrogates the harms of both legal and illegal activities (see Sollund 2019 among others) and the blurred line between what is defined as legal or illegal, often due to the power dynamics of who determines illegality (South 2007; White 2011). As indicated, in the following sections we will provide evidence that the convergence of wildlife and drug trafficking is a source of violence to wildlife and to the environment.

## Overviews of the illegal wildlife and drug trades

Drug trafficking and smuggling have been the subject of extensive research with various descriptions of participants and estimates of the value of the global illicit market as worth up to $652 billion per year (GFI 2017: xi). Although there has been a significant increase in research devoted to wildlife trafficking this has nonetheless been relatively limited. This may reflect persisting biases in global law enforcement policies as well as the lower estimated value of this criminal trade as worth about $5 – $23 billion without inclusion of fish and timber (which would be an additional $67–193 billion) (GFI 2017: 99). Of course, both sets of estimates about value will be based on assumptions and may also reflect different points of time or transactions along the supply chain (e.g., Andreas and Greenhill 2011; Mackenzie 2020). For example, a UNODC (2011) report estimated cocaine production earns the farmers $1 billion, whereas the final gross profits are around $85 billion. Similarly, the collector of a falcon may earn a few dollars, but they can be sold for over $10,000 (Wyatt 2009). However, the dark figure of both illegal markets is notoriously difficult to uncover and, by definition, cannot be measured by official economic indicators, but only by estimates of what these might be.

Whilst monetary estimates might be helpful in highlighting the suspected scale of an illegal market, they remain problematic because of their simplicity, which never captures value - intrinsic, aesthetic, cultural and so forth - other than financial. This seems particularly the case with wildlife trafficking, where living beings are reduced to vessels and commodities, and their own lives and autonomy ignored. Thus, estimations grounded in profits, criminal or otherwise, hide the environmental destruction integral to achieving the profit margins. Illegal drug cultivation is a source of deforestation, biodiversity loss, water shortages and pollution stemming from the use of pesticides to grow the drug crops as well as the chemicals used to manufacture the drugs (Burns-Edel 2016). Pollution that destroys ecosystems and harms biodiversity and individual wildlife also arises from law enforcement efforts to eradicate illicit crops through aerial fumigation (Rodriguez 2004). Likewise, the illegal wildlife trade (and an unsustainable, parallel legal business) is a source of deforestation and biodiversity loss, while demand for live wildlife and wildlife products is met through use of techniques of abduction and killing (Wyatt 2013; van Uhm 2016; Moreto and Pires 2018; Petrossian 2019; Sollund 2019). The environmental harms of both trades are therefore incalculable, global and complex.

Similarly, the illegal entrepreneurs engaged in these trades and markets, have become more globally mobile, traversing international borders, and operationally complex, infiltrating new illicit markets or expanding existing ones (Aas 2013; Varese 2011). New collaborations, alliances and fluid criminal networks are developing around the world influenced by improved infrastructure in faraway regions and expanded communication opportunities (Morselli 2009; Galeotti 2014). In this context, the UN (2003: 12) stated that ‘traffic of endangered species frequently occurs in connection with the illegal trade of other products or substances such as drugs’. Europol (2011: 40-41) added that the trade in endangered species ‘is of increasing interest to poly-criminal organised crime groups. Groups involved in high-level drugs trafficking in Brazil, Colombia and Mexico have established a notable role in the illegal supply of endangered species to the EU and US markets’. Interpol (2018: 2) echoes that ‘wildlife trafficking networks - across land, sea and air - are also commonly used to smuggle other illicit commodities, such as drugs’.

These trades are global, but with clear regional trends. For example, there is global demand for cocaine from Latin America, but the primary smuggling destination has been the US; forms of cannabis are produced and trafficked more widely on a global basis; opioids and amphetamines see high demand in North America and Oceania; Europe, and in particular Eastern Europe, is a primary destination for opioids. Regional trends related to wildlife trafficking and markets include the culturally driven demand for ‘traditional’ medicines in China in particular, but across Asia generally, while demand for live pets and dead animals feeds into fashion industries serving Europe and the US (Wyatt 2013; van Uhm 2016). (Il)legal logging and timber transport fuels a continually growing market, despite awareness of the consequences of deforestation, with consistently high demand being associated with China, the US and Europe (Seneca Creek and Associates 2004; Boekhout van Solinge et al. 2016). This illustrates that wildlife trafficking is a diverse illegal market and the intersections with the drug trade are equally diverse in an increasingly globalized world.

## Purpose and methodology

Reuter and O’Regan (2017: 7) claim that several sources of information referring to connections ‘between animal smuggling and other criminal organisations’ come from organisations with ‘neither authority nor objectivity’. In this article, we draw on a range of sources including reports from Interpol, Europol and the UN, which are seen as authoritative, although we acknowledge that police and (inter)governmental agencies, like most everyone, operate with an ‘agenda’. In addition, we scrutinize academic articles and official reports by governmental agencies that were available and accessible by using Google Scholar and our University library catalogues. Specific keywords, such as ‘drugs and wildlife’, ‘wildlife and narcotics’, or ‘animals and cocaine’, among others, were used to retrieve the information.

We also draw on original fieldwork carried out by the first author in Colombia, Laos and Myanmar (Burma) during 2017–2020. The goal of this on-going study is to understand the diversification of organized crime into the illegal trade in natural resources, by looking at the convergence between environmental crime and other serious crimes, including wildlife and drugs. At the time of writing, 67 study participants had been interviewed during fieldwork, including representatives of local communities, NGOs, and officials, as well as people directly involved in the illegal wildlife trade such as poachers, smugglers, intermediaries, traders, and suppliers. Study participants were identified using convenience sampling in all three countries and were approached due to their familiarity or involvement with the illegal wildlife and drugs trade. The participants were recruited through snowball sampling, which is useful in contacting members of a population who are difficult to access. According to Polsky (1967), use of referrals is an effective strategy to recruit participants from the underworld and help the researcher manage any risk associated with new contacts as well giving the interviewer some level of verification and trustworthiness. Therefore, future informants were recruited from among the acquaintances of participants and through the first point of access (Goodman 1961). Interviews attempted to tap into the participants’ knowledge of the various modus operandi, network structures and the convergence between the illegal wildlife trade and drugs, among other topics.

The semi-structured nature of the interviews afforded the ability to probe for additional information, when necessary (Davies et al. 2011). Most interviews were conducted in the local languages, therefore a local interpreter regularly assisted with translation. The interviews were conducted in various settings, including bars, restaurants, casino’s, shops and in the homes of interviewees. The length of the interviews varied between 30 min to 2 h (in exceptional cases). As in other sensitive research on perpetrators, victims or witnesses of crimes, anonymity and confidentiality play fundamental roles in developing a relationship with informants (Noaks and Wincup 2004). The identities of our study participants are concealed, or pseudonyms are used to protect participants against adverse effects and criminal prosecution. No financial compensation was provided for the participants involved, but sometimes small gifts were offered as a symbolic way to thank the respondents. Where possible, interviews were digitally recorded, but informants regularly asked not to have the interviews recorded. In such cases, comprehensive note taking ensured the recording of the data. This also resulted in more trustful situations where people were able to speak in more detail about the criminal aspects of their lives (Polsky 1967). In addition to interviews, useful information was obtained through informal discussions and direct observations in the research locations, including wildlife markets. Such information was valuable in further contextualizing the data obtained during the interviews, as well as corroborating or refuting information derived from respondents (DeWalt and DeWalt 2011).

Initial or open coding was first performed in NVivo. Initial coding requires the careful examination of separated sections of transcribed data in order to identify similarities and differences (Davies et al. 2011). To uncover overarching theoretical concepts, pattern coding was conducted on the data. Pattern coding can be employed to find patterns or relationships (Maxwell 2005). The following theoretical concepts emerged: combined contraband (1), camouflage (2), multiple trade lines (3), shared smuggling routes and transportation methods (4), barter trade (5), and laundering drug money (6).^2^ All categories were identified in both the literature review and the interviews; therefore, those concepts are the basis for the development of the themes that will be discussed in this study.

Our research has several important limitations. First, the analysis of the empirical data sheds light on the naturalistic and empirical reality of the regional case studies. Although these are limited in geographical scope, the literature research is international in breadth. Second, the fieldwork was completely dependent on the availability of participants: while in some situations, recruitment was straightforward, in others it was difficult to meet the right people who were willing to be interviewed. Third, the role of interpreters can be seen as a limitation regarding the accuracy of their translation (Noaks and Wincup 2004). However, it was a great advantage that interpreters could help to clarify information in the context of the sociocultural backgrounds of the participants.

## Results: Overviews of trafficking motives and methods

We note that there are varied motives for smuggling a commodity and reasons for using particular techniques (e.g., smuggling illegal commodities mixed together, or smuggling one illegal commodity hidden within or by a different but legal commodity). Our analyses do *not* suggest that wildlife and drugs trades and smuggling are now converging ‘always and everywhere’. They do suggest that when this happens it often appears to occur in ways that we categorize as follows: combined contraband (both illegal wildlife and illegal drugs in shared shipments), camouflage (legal wildlife-illegal drugs in shared shipments), multiple trade lines (illegal wildlife and illegal drugs, where the groups run different businesses), shared smuggling routes and transportation methods (illegal wildlife and illegal drugs using the same pathways, but not at the same time), barter trade (exchanging wildlife for drugs and vice versa), and laundering for drug money (legal wildlife industries as a cover for illegal drug money) (Table 1).

**Table 1:** Overview of trafficking methods and motives

Method	Motivation	Description
Combined contraband	Mixing illegal commodities may be because traffickers want to smuggle both for reasons of convenience, expedience or opportunity	Illegal wildlife and illegal drugs in shared shipments
Camouflage	A legal commodity (in this case wildlife) is used as a vessel for smuggling/hiding the contraband	Legal wildlife used to hide illegal drugs
Multiple trade lines	Spread risks and increase control (and profit) by dominating multiple trade lines and routes	Illegal wildlife and illegal drug lines and routes controlled by the same network/group
Shared smuggling routes and transport methods	Smuggling multiple commodities because the access to smuggling routes, smuggling methods and/or paid corridors makes these routes profitable and multi-purpose	Illegal wildlife and illegal drugs being smuggled along the same route, but at different times
Barter trade	Cashless transactions that are very hard to trace facilitate illegal trades simultaneously	Illegal wildlife in exchange for illegal drugs (and vice-versa)
Laundering drug money	Making illegally-gained proceeds from drugs appear ‘clean’ by use of a legitimate wildlife company	Legal wildlife industry used to launder the proceeds from illegal drugs

### Combined contraband

Mixing *illegal* commodities together may occur for reasons of convenience, expedience or opportunity. In ‘Smuggling Multi-Consignment Contraband’ (MCC) Lichtenwald et al. (2009: 18) state that when ‘the smuggling network is already established; certain types of contraband combinations do not raise the level of detection, and changing geopolitical and market pressures make MCC a reasonable business decision’. Already in the 1990s and 2000s, reports claimed that Colombian drug cartels^3^ were smuggling ‘mixed shipments of drugs and wildlife products’ into the US (UN 2002: 6). Elliott (2009: 66) provides several examples of combined contraband, including protected turtles that ‘have been found in the same shipments as marijuana’ and ‘parrots and drugs [that] have been smuggled together from Cote d’Ivoire to Israel’, while Phillips (2010) reports that in 2010, 285 radiated tortoises and 15 spider tortoises were smuggled from Madagascar to Malaysia in two suitcases that also contained several kilos of cannabis. More recently, Wyler and Sheikh (2013: 4) refer to ‘mixes of narcotics and wildlife [that] include cases involving elephant tusks stuffed with hashish and exotic parrots smuggled with methamphetamine pills’. Another aspect of combined contraband, according to Interpol (2015: 6), is to ‘use …[illegal] timber to conceal the drugs, including in hollowed out logs’.

According to interviewees in Myanmar (Burma) and Laos, a recent trend of combined contraband is pangolin scales smuggled together with methamphetamine into China.^4^ One important wildlife trader near the town of Kengtung described the criminal infrastructure used to get wildlife into Mong La and then smuggle it across the border to Daluo in China as also providing opportunities for drugs smuggling. He explained that sometimes methamphetamine is hidden inside pangolins which not only increases the profit, but also makes being stopped for drug checks less likely along the way. An interviewed undercover agent in Bangkok echoed this, recalling an investigation that involved a car used for methamphetamine-smuggling across the Thailand-Laos border also being used for smuggling pangolins.

Similarly, interviewees in Darién, a remote, road-less swath of jungle on the border of Panama and Colombia, confirmed that cocaine is hidden inside illegal timber and the same logistics are used for reasons of convenience; the contraband is transferred by river to the first villages with access to the road. Once they reach a small Panamanian town, Yaviza, where timber permits can be ‘obtained’, the illegal timber is effectively ‘laundered’ and becomes ‘legitimate’. Then, both the (formerly illegal) timber and the cocaine can pass control points, showing the legitimate paperwork for timber along the way. This illustrates how different operators in legal and illegal markets can cooperate for mutual benefit from facilitating the movement of combined contraband.

### Camouflage

Further evidence of the interface between legal and illegal markets comes from instances of *legal* wildlife being used to smuggle illegal drugs. In such cases, smugglers may use their legitimate infrastructure to camouflage contraband by mixing it with legitimate goods (Block and Chambliss 1981). Different methods of such camouflage, now well known, can be employed at different stages of the processes of cultivation, production, capture or collection and many narcotics groups have used legal wildlife as a cover in the cocaine trade. For example, legal timber has been used to camouflage the transport of cocaine, heroin and methamphetamine (Devlin 2016) and cocaine has also been hidden in doubled-up bags of hundreds of tropical fish (BBC 2012). In the latter case, the offenders placed a small plastic bag with dissolved cocaine within a larger bag full of tropical fish and water (BBC 2012). Thousands of fish were killed during a trial run without the cocaine, where the fish were left to suffocate and thousands more fish were killed whilst smuggling the cocaine. This case highlights the harm to non-human animals prevalent in trade and trafficking. There are also documented instances of parrots, sharks and boa constructors being filled with cocaine, which depending upon the species, may be legally traded. Sina et al. (2016: 33) add,

‘In some cases, snakes, alligators and reptiles are not being used as commodities in themselves but simply as a form of concealment. In such cases, therefore, the animals are regarded not as an additional source of profit but as a means of ensuring that the drugs themselves are not seized and provide the profits envisaged’.

Study participants referred only incidentally to the camouflage method of using wildlife to hide drugs, and it was not mentioned as a common method. Perhaps this is due to the sample of respondents in the study; many interviewees were involved with high-value wildlife such as tiger, elephant and rhino products, which are trafficked rather than legally traded. However, interviewees did provide some examples and notably, an expert from Interpol and a representative of an environmental NGO in Bangkok, both highlighted that legitimate trade in animals or animal products could be used to hide drugs and distract customs officials. There are a number of cases where legal trading has indeed been used as a cover in Thailand (van Uhm and Nijman 2020). More often it happens that timber is being used as a cover for drugs, as explained by participants in Acandí and Turbo in Colombia. Both illegally and legally harvested tropical timber has been used to smuggle cocaine hidden in the logs across the borders to avoid inspections.

A wildlife crime expert in Laos explained that dangerous ‘wild’ animals are also employed to protect stores of drugs as a means of discouraging discovery by officials. For example, in the 1990s, there were several cases of drugs being trafficked together with dangerous snakes to confuse sniffer dogs and scare customs officials (Chiszar et al., 1992). A participant from TRAFFIC North America reported: ‘you have cases where there are drugs hidden in false compartments within crates containing live venomous snakes and written on top it says: “Venomous snakes. Don’t open!”’

### Multiple trade lines

In addition to combining and camouflaging shipments, crime groups can run multiple trade lines simultaneously. A study by RUSI (2019), based on datasets from different law enforcement departments of one East African country, showed the significance of such examples of convergence: ‘[I]n nearly a hundred contact points just in the wildlife data, we found convergence of about 3/4 with the narcotics sector (…) The networks are moving everything’. For example, in 2018, members of a drug running organization from Kenya pleaded guilty to drug trafficking charges, including the trade of large quantities of heroin and methamphetamine.^5^ They were also linked to around 30 tons of ivory seizures. In a recent indictment in 2019, members of another African criminal enterprise were charged with large-scale trafficking of rhino horns and elephant ivory valued at more than $7 million, plus heroin distribution and money laundering. ‘The suspected criminal masterminds not only conspired to traffic huge amounts of heroin to New York, but also directed a multimillion-dollar poaching scheme to traffic in rhinoceros horns and elephant ivory’.^6^ In Europe, as a result of investigations into Vietnamese crime groups, the on-going Operation Osseus in the Czech republic, has revealed multiple illegal trade lines, involving movement of synthetic drugs (methamphetamine etc.), tiger bones, ivory and rhino horn from Africa.^7^ According to Nožina (2020 and in Shelley and Kinnard 2018: 126) ‘[r]hino horn shipped through the Czech Republic converges with many other forms of crime including illicit trade in consumer goods, methamphetamines, and marijuana’.

In line with the investigations above, interviewees in Myanmar (Burma) explained how rich Burmese methamphetamine lords in the town of Kengtung in East Shan State, known for its opium cultivation and methamphetamine production, became involved in multiple trade lines. Besides sales of methamphetamine, they pursue profitable additional sidelines in tiger skins and bones for tiger-bone wines. Since tiger skins and wines are highly profitable and worth around $8000–9000 per skin and up to $1000 per wine bottle respectively on the local market, the investment required to enter this trade is only available for a few: the rich layers of Kengtung, who often have well-established relationships with local governmental officials. This shows that the trade in valuable wildlife products in such local political economies can be linked to money previously earned through other illegal businesses, which in Shan State in Myanmar is related to the historical and socioeconomic importance of drug trafficking.

As observed by the first author, another example is the Special Economic Zone (SEZ) in Laos where high-value wildlife contraband such as tiger-bone wine, casques of helmet hornbills and rhino horns are being sold openly alongside drugs, including *yaba* (a synthetic drug based on methamphetamine and caffeine). In particular, the Kings Romans Casino is a noteworthy market venue, where rhino horns feature among many other illegal goods. In 2018, the United States Department of Treasury (USDT) placed the Kings Romans Casino on its organised crime sanctions blacklist, calling it a transnational criminal organisation engaged in child prostitution, and human, drug and wildlife trafficking. Interviewees, including two employees of the Kings Romans Casino, explained that the organisation stores and distributes both methamphetamine and illegal wildlife products, including tiger bone wines and rhino horns. Large consignments of methamphetamine seized in Thailand, and illegal wildlife trade from neighbouring countries as well as Africa, have been traced back to Kings Romans (van Uhm and Wong 2021). According to illegal wildlife traders in the SEZ, the owner of the casino not only collaborates with local officials, but he also has agreements about the trade; for example, about percentages of profit paid to officials, what wildlife or drugs are offered for sale in the casino and when governmental inspections will take place. This reflects the social and power dynamics between the multifaceted criminal groups, the wildlife and drug traders and the government officials.

Furthermore, Wagner et al. (2020) found in Mexico that the drug cartels in the state of Chihuahua run illegal logging operations. The illegal timber is laundered into the legal supply chain entering the US. Similarly, according to an interview with a governmental official from Jakarta, a convicted Indonesian drug lord was linked to a pangolin trafficking network over a five-year period. The investigation showed a financial flow of around $6 million from convicted drug dealers. The crime group used company accounts to co-mingle revenue from a legal fishing company, and illegal proceeds from pangolin and drug trafficking. This illustrates how illegal profits from one crime (drug trafficking) are used to support further illegality (wildlife trafficking), reflecting the creativity and flexibility to establish multiple trade lines.

### Shared smuggling routes and transport methods

Crime groups and networks are sometimes involved in profiting from sales of both drugs and wildlife and will employ the same drugs and wildlife smuggling routes and transport methods for both (Europol 2015; UNEP 2016). Already noticed in the 1990s and early 2000s, access to smuggling routes, smuggling methods or corruption established for drugs may facilitate other forms of crime (van Duyne 1995; Naylor 2005), including wildlife trafficking. For example, in 2006 US Customs discovered five tons of ivory in a sea container from Cameroon along with traces of narcotics. Officials stated that the crate had also been used to ship several different kinds of contraband, including drugs (Nelson 2020; Lichtenwald et al. 2009). The outcomes of a study by the US Office of the Director of National Intelligence (ODNI) show that ‘criminal [drug] networks often test routes and methodologies with wildlife products before using them for higher-stakes criminal activities’ (TNRC 2020).^8^ Similarly, UNODC (2016: 50) states that ‘[r]ecent seizures of ivory and rhino horn would indicate that criminal syndicates are adopting concealment methods for wildlife products that are normally associated with drug shipments’. In Europe, Europol (2011: 41) echoed that ‘the concealment methods developed for drug trafficking are now used to traffic endangered species’.

Indeed, the smuggling infrastructure of trafficking may be attractive for different commodities. In Shan State, wildlife traders explained how established and successful smuggling *routes* between Tachileik, Mong Lin, Kengtung and Mong La are more important than the commodity itself. Such ‘routes’ (in reality a logistical operation to enable movement of goods along a chain) use a cover (like fruits or vegetables) to hide the illegal goods - whether tiger products such as bones and skins, or methamphetamine - and bribe officials or militia along the way. Similarly, smugglers of drugs from Laos via the Mekong River into Thailand were said to also be involved in smuggling pangolins along the same routes. Wildlife was then smuggled in the other direction, via Laos into Vietnam, according to a wildlife expert in Vientiane. They may smuggle whichever product for illegal gain, from wildlife to drugs, and are ‘not necessarily acting out of ideology when moving wildlife along the smuggling chain’ (Wyatt 2013: 85).

As in the Laos-Vietnam example above, an often-noted element to shared smuggling routes is that drugs may move in one direction and wildlife in the other along the same route using the same transportation. In the 1980s and 1990s, this ‘backloading’ was a serious issue in relation to birds and reptiles worth up to $80 million a year in Australia. Animal backloading was familiar to Australian wildlife officials in the following way:

‘A smuggler that brings a load of drugs into Australia often prefers to make the return trip equally profitable. One way to accomplish this is to backload relatively small but rare animals that will fetch high prices in the U.S., Europe or elsewhere’ (Chiszar et al. 1992: 7).

Similarly, organised crime groups do not directly run all illegal activities, such as drug, human and wildlife smuggling, but control smuggling routes. For instance, interviewees explained how illegal entrepreneurs need to ask permission and pay tax to the Gulf Clan, known for cocaine trafficking, to cut trees and transfer timber to a transit hub via the Cacarica river in Colombia (extortion to be paid is around 15%), illustrating the role of established paid corridors (van Uhm 2020). Thus, crime groups exploit the established trade routes and modus operandi used to traffic all kinds of contraband and, consequently, ‘drugs (…) are uncovered as part of environmental crime operations’ - or the other way around (Interpol 2015: 6).

### Barter trade

A clear manifestation of the interface between wildlife and drugs as illegal commodities appears when they are exchanged for one another. For example, in the 1990s, Colombian drug cartels would exchange drugs for endangered species resulting in cashless transfers (Kazmar 2000) and planeloads of smuggled birds from Australia were being exchanged for heroin in Bangkok, with the drugs then being exported to Australia (Cook et al. 2002). In South Africa, crime groups have reportedly provided highly-prized (but illegal) catches of abalone in exchange for methamphetamine (‘tik’) (Steinberg 2005). The relations of exchange varied from Chinese crime groups bartering methamphetamines for abalone, forging cash-free trade relationships with Cape gangs that control the local drug market, to poachers bartering abalone for drugs for resale or, alternatively, for their own consumption (Brick et al. 2014; Oakes et al. 2018). A reported leader of one of the gangs on the Cape Flats, South Africa, stated that ‘he could trade $43,000 worth of abalone for methamphetamine worth $64,000’ (Goga 2014: 4). Even where wildlife trades appear to be mono-specialist in orientation they do not ‘stand-alone’ as if separated from their socioeconomic and cultural contexts and the related or parallel, legal and illegal, trades that may be occurring.

For example, cashless transactions are very hard to trace and therefore useful to facilitate illegal trades. In the jungles of Latin America and Southeast Asia, a similar connection can be found between the illegal trade in timber and the drugs trade:

‘Timber traffickers, who have built a huge logistical network for bribing officials and moving illegally harvested wood out of the country, are now working with drug traffickers to move drugs through the same trafficking routes (…) drugs are used as payment in exchange for high-value timber species that have been illegally harvested’ (Interpol 2015: 6).

This is confirmed by interviewees in Panamanian jungle villages in Darién, who explain that it is often more convenient to exchange wildlife, including timber, instead of selling them, in particular where the local economy has an abundance of wildlife and drugs, while money is scarce.

### Laundering drug money

Several reports associate wildlife trafficking with money laundering (UNODC 2010; Europol 2013), but wildlife trading may also be a cover to launder illegal money from other serious crimes, such as drug offences (van Uhm 2018; Interpol 2016). One of the first major drug-wildlife crime cases in history, uncovered through Operation Cobra^9^, involved the head of a drug syndicate combining his cocaine trade with an exotic animal business in Miami. The animal business turned out to be a perfect cover for drug trafficking; they used codes such as ‘I need three cockatoos’, meaning three kilos of cocaine or ‘three lesser- crested cockatoos’, which meant three ounces (van Uhm 2018). The case illustrates how the syndicate used the *legal* wildlife enterprise to launder drug money through several investments, false loans and an offshore corporation.^10^ It also demonstrates that our categories are not discrete as the syndicate also used the business to hide drug trafficking (camouflage) and wildlife trafficking (multiple trade lines). The head of the syndicate stated in an interview: ‘Back then, I sold drugs to maintain my animal habit. I got to a point where I was on the phone saying Mario’s Drugstore, specializing in marijuana, cocaine, and quaaludes. Anybody interested come and get it.’ A former employee explained: ‘Pretty much any way you would smuggle illegal drugs, they would do the same with the animals, but the penalties for that were far less than if you were busted for smuggling drugs’.^11^

In another case, an agreement with an owner of a timber company provided a perfect cover to launder money from the sale of drugs. The owner of the timber company received the proceeds of the drug sales and then provided the drug dealers with legitimate cheques, which were placed into personal accounts. The illegal money was mixed with legitimate funds from the timber and the owner received 13% of the laundered money (AUSTRAC 2010).

Law enforcement officials in Yaviza (Panama) and Turbo (Colombia), on the outskirts of the Darién jungles, explained how profits from cocaine can be used as investments for legitimate timber companies. Some local timber entrepreneurs have become rich from coca trade in the region and in the past few years, a number of important cases in Panama have come to light. For instance, the owner of a Panamanian timber company received a percentage of profits from cocaine trafficking from his brother for laundering the proceeds via his company in Yaviza. On the Colombian side, an environmentalist in Triganá explained how illegal drug money from members of the notorious Gulf clan was laundered through legitimate timber and gold companies. The embeddedness of cocaine in the region meant that it was inevitable that some of the timber investments originated from the illegal drug trade and were the result of planned money laundering.

## Discussion

As noted earlier, a prima facie question to consider in relation to illicit markets might be why some actors appear to remain focused on a single commodity, whilst others engage in various ways with other markets? It would be foolish to suggest that a definitive answer to this can be offered here, but the forgoing should illustrate that it is not possible to separate markets from their socioeconomic and cultural contexts and hence openings to related or parallel, legal and illegal trades. Recognising the advantages of cooperation or perceiving the promise of increased profit offered by other opportunities might provide the basis for convergence or divergence as discussed further below.

### Convergences

Illicit markets are complex and the examples of activities and transactions that are provided above illuminate some of the different dimensions of trades and trafficking involving drugs and wildlife. One reason for a criminal enterprise to remain focused on wildlife markets and trafficking may be that they present a low risk with the possibility of medium to high rewards. Wildlife trafficking is profitable on its own. However, as it has previously received relatively little attention from customs, border agencies and police, this may mean that wildlife (including fish and timber) can provide tempting opportunistic hiding vectors for smuggling of other commodities. As wildlife trafficking becomes the focus of greater attention from police and security agencies (Duffy 2016), the low-risk character of this business may be diminishing. At the same time, the attraction of possibly greater reward from smuggling drugs might influence decision-making about the mixing of commodities (Shelley 2018; Mackenzie 2020).

This trend and explanation would reflect the emergence and engagement of multifaceted crime groups in wildlife trafficking that are also involved in other serious forms of crime (van Uhm and Nijman 2020; van Uhm and Wong 2021). This illustrates the dynamic structure of some of the crime groups that are able to diversify into new forms of crime in order to adapt to socioeconomic, political and ecological circumstances. Cases examined in our analysis illustrate forms of *convergence*, but it should be noted that connections could also be shown by considering cases of *divergence* between the trades.

### Divergences

Below we give several examples of the *divergence* (rather than convergence detailed above) of criminal groups from drugs to wildlife trafficking. In a famous illegal wildlife case, investigation *NOAH* (March 1996/Interpol), one of the main suspects, a Dutch reptile smuggler with a global network, was previously involved in smuggling drugs, as were other wildlife traders exposed in this particular investigation (Interpol 1996). Interpol (1996: 67) stated in one of their conclusions to the investigation that: ‘highly organized criminal groups such as [this] organization are implicated in the illegal trade because the low penalties imposed for offences make traffic in animals an attractive alternative to traffic in other illegal substances’. Similarly, in Daluo and Mong La, two Chinese-Burmese border towns, smugglers explained how they gradually became involved in trafficking elephant skins, pangolin scales and tiger bone products influenced by internationally agreed, repressive drug policies, which illustrates the significance of geopolitical influences on diverging criminal careers (van Uhm 2019). Other examples of divergence may include drug, contraband and motor vehicle traffickers who entered illegal rhino and elephant markets in recent years (Rademeyer 2012; Hübschle 2016).

These factors have remained relatively constant in recent decades, although the dynamics of specific trades and transactions can change when the value of the wildlife commodity increases. For instance, the leader of a Mexican drug-trafficking organization was murdered for failing to pay $1 million for a shipment of rare and expensive bladders from the *totoaba* fish destined for Asia. In this context, Mexican officials believed that the Mexican drug cartels in the Baja area of Mexico were shifting their activities into wildlife smuggling through the US and onto China (Felbab-Brown 2017; Martínez and Martínez 2018; Arroyo-Quiroz and Wyatt 2019).

## Conclusions

There is, as always, a need for more empirical data collection examining any links between these two - and other - illicit markets. As Sosnowski (2019: 8) remarks, ‘Illicit markets are estimated to represent a fifth of global economic activity, but many remain poorly understood due to their clandestine nature’. Further analysis would consider what lessons can be learned and whether there are synergies in enforcement and conservation efforts that need to be explored. Such research should not under-estimate the significance and embeddedness of commodity chains in contemporary globalised markets reflecting a shift from social and economic arrangements as ‘bounded institutions and places’ to a world of ‘mobilities and interconnectedness’ facilitated via ‘networks, flows and spatiality’ (Jackson et al. 2004: 10; Castells 2000; Urry 2010; South 2007: 242–3). The participants in these activities are agents and organised groups – a diverse range from significant but single entrepreneurs (Haller 1990) to interacting criminal groups (Bruinsma and Bernasco 2004; van de Bunt et al., 2014) to global but disarticulated international networks that ‘offshore’ the financial value produced by local actors (Murphy 2019).

As Aziani et al. (2019) argue, while there may be ‘distinct foci’ to different theories of organised and transnational crime based on either ‘rational decision maker’ approaches (‘illicit commodities and services are supplied because it is profitable do so’) or on the ‘social embeddedness’ perspective (emphasising the importance of social relations as integral to the ‘dynamics of criminal organisations’), these views need not be regarded as oppositional and incompatible. The business trading choices and strategies discussed here are complex in terms of factors such as individual decision-making and organisational properties and dynamics and should be understood in their local political economy. Future research needs to unpack some of this intricacy, perhaps employing, as Huisman (2016: 55) suggests, a mix of ‘life-course criminology’ and ‘organisational life-cycle models’, and paying attention to the influence of externalities such as law and enforcement (with ‘positive’ effects for society, ‘negative’ effects for criminal trade participants). This is not, however, a world that can be understood solely in *economic* terms.

For future analysis, we must remember Sollund’s (2020) point that to contemplate forms of trafficking – of humans, animals, or (we would add) drugs, − solely ‘in economic terms’ is to lose sight of associated and underpinning ‘pain, suffering, and death’. Wildlife trafficking as a ‘business’ does not take place in isolation from other crimes and harms, as we have demonstrated with the connection to drugs, although there are different and real harms affecting the sentient, pain-feeling, non-human species that are subject to trafficking who suffer in awful ways. Thus, any future analysis should include the cultural elements underpinning trafficking.

Nonetheless, and unsurprisingly, it is the potential for health harms to humans through transmission of zoonotic viruses that is most likely to not only lead to enhanced efforts to prevent the continuation of illegal wildlife markets, but also change the very nature of the markets due to lockdowns, travel restrictions and changing consumption behaviour following the pandemic (Wildlife Justice Commission 2020). In December 2019, the emergence of the Coronavirus in Wuhan, China, was picked up in the local media by the regional office of the World Health Organization (WHO 2020). The novel nature of the virus and its rapid global spread led to renewed international recognition of the links between legal and illegal trades in various species and pathogen pandemics. Examining the convergence between trades in wildlife and drugs helps us to understand how markets interact, which is relevant for responses from the worlds of both policy and law enforcement, as well as public health and medicine. The urgency of response to zoonotic virus transmission will mean calls for, in particular, the end to illegal wildlife trade, markets and consumption. In pursuit of this, looking to the lessons to be learned from the long history of attempts to prohibit drug trafficking, markets and consumption will be advisable - although not necessarily encouraging.

## Footnotes

1 We are aware that there is now an enormous literature on illegal markets and trade, but for reasons of space we cannot include reference to all relevant studies here. For overviews see e.g., Kemp and Galemba (2020).

2 Van Uhm and Nijman (2020) connect the different categories to the environmental crime continuum in their article ‘The convergence of environmental crime with other serious crimes: Subtypes within the environmental crime continuum’.

3 For instance, in the 1980s, the drug lord Pablo Escobar from the Medellín cartel started illegally flying endangered species to Hacienda Nápoles, as he called his 530 hectares of jungle and grassland along the Magdalena River in Colombia. Pablo Escobar loved animals, in particular birds, explained his brother Roberto Escobar during an interview at his villa. He sought expert advice on which species could survive in this location and used military transport planes after opening hours to land the animals at the airport of Medellín.

4 See also Jacobs et al. (2019) ‘Myth debunked: Keratinous pangolin scales do not contain the analgesic tramadol’. (The reason for this combination of contraband is unclear although rumours were reported that pangolin scales were being used as a drug substitute for methamphetamine, and also that drug networks have adopted this new commodity into their portfolio).

5 United States v. Ibrahim Akasha Abdalla, a/k/a “Ibrahim Akasha,” et al., S12 14 Cr. 716 (VM); United States v. Baktash Akasha Abdalla, a/k/a “Baktash Akasha,” et al., S11 14 Cr. 716 (VM).

6 Indictment United States v. Moazu Kromah et al., 19 Cr. 338 (June 13, 2019).

7 Czech enforcement authorities, ‘*Trade in rhino horns in the territory of the Czech Republic’,* 2.

8 See https://www.worldwildlife.org/pages/tnrc-blog-understanding-crime-convergence-to-better-target-natural-resource-corruption.

9 no 87–928-Cr-KEHOE / 16-12-1987

10 United States District Court, Southern District of Florida, ‘Operation Cobra indictment’ (16 December 1987).

11 Tiger King, Netflix 2020, Episode 2–11 min, 5 s.
